# Supplementary material for: Genome-Wide Characterization of Effector Protein-Encoding Genes in Sclerospora graminicola and Its Validation in Response to Pearl Millet Downy Mildew Disease Stress
Source: J Fungi (Basel). 2023 Mar 31;9(4):431. doi: 10.3390/jof9040431 (PMC10142805; doi:10.3390/jof9040431)
Supplement: Supplementary file 1 [file jof-09-00431-s001.zip › jof-2241602-supplementary.pdf]

Phylogenetic relationships RxLR protein other proteins

Effector Protein	Protein identity	Organism	Identity %	E-value	Genbank accession ID
8311_g	Putative secreted RxLR effector protein	*Phytophthora cinnamomi*	25.75%	2e-09	KAG6615993.1
60945_g	Putative secreted RxLR effector protein	*Phytophthora cinnamomi*	26.09%	5e-11	KAG6615993.1
35983_g	hypothetical protein PsorP6_016330	*Peronosclerospora sorghi*	28.14%	2e-07	KAI9906962.1
6877_g	hypothetical protein PsorP6_014941	*Peronosclerospora sorghi*	31.51%	7e-06	KAI9909160.1
60741_g	putative RxLR effector	*Phytophthora cinnamomi*	28.21%	6e-26	KAG6599847.1

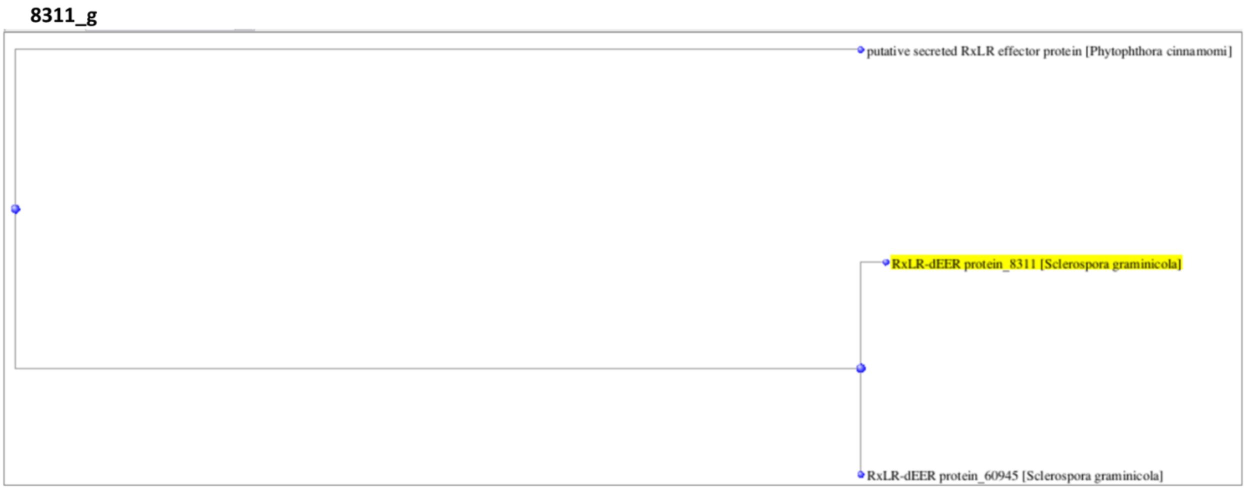

Figure S1. Phylogenetic tree of protein 8311_g

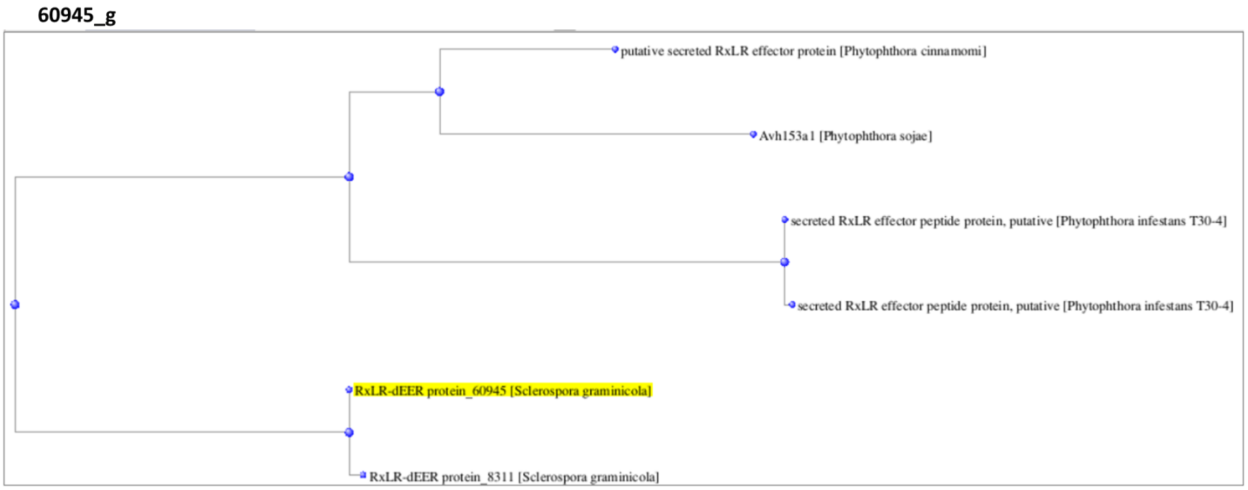

Figure S2. Phylogenetic tree of protein 60945_g

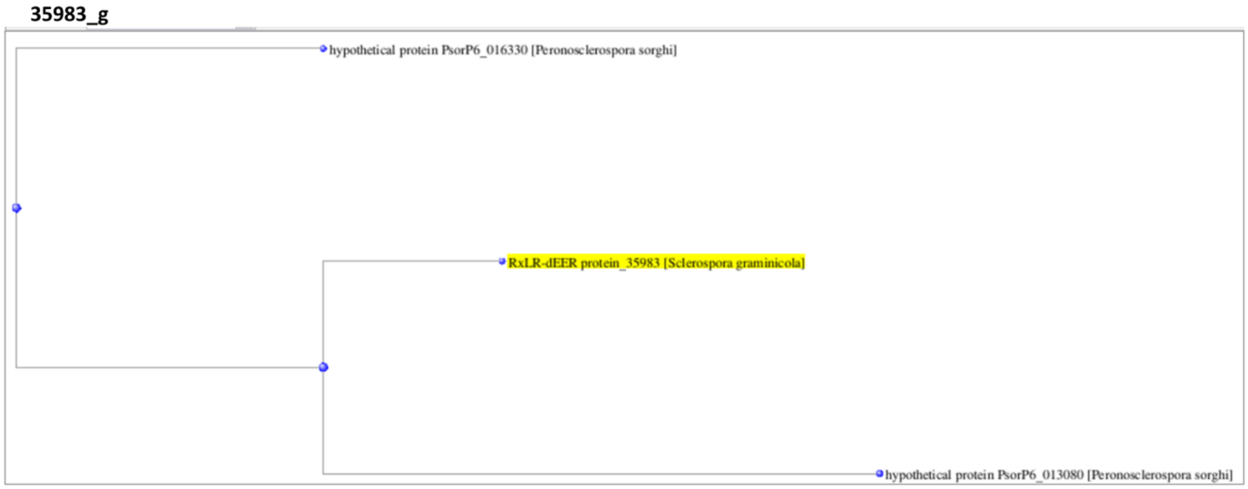

Figure S3. Phylogenetic tree of protein 35983_g

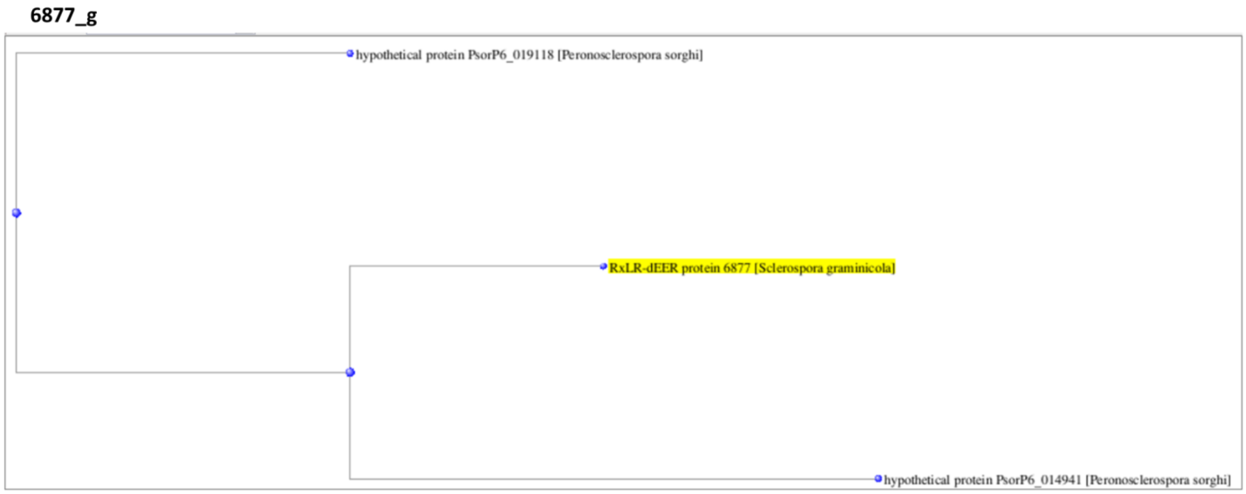

Figure S4. Phylogenetic tree of protein 6877_g

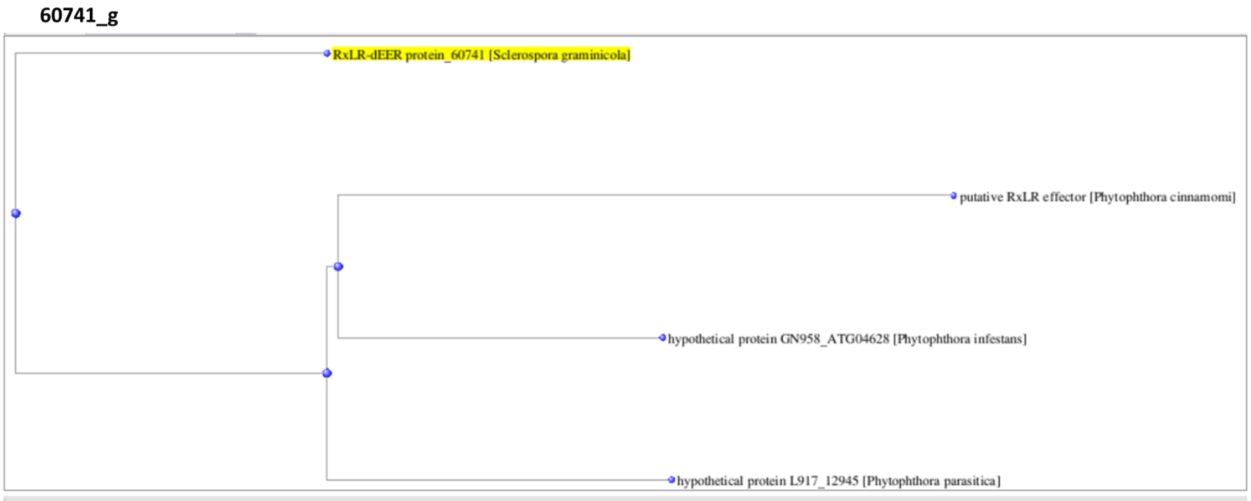

Figure S5. Phylogenetic tree of protein 60741_g
